# Long term efficacy of prosthetic inguinal herniorrhaphy concomitant with robot-assisted laparoscopic radical prostatectomy

**DOI:** 10.1007/s00345-023-04625-3

**Published:** 2023-09-27

**Authors:** Nelson Peixoto, Elisabeth Grobet-Jeandin, Fabian Schoofs, Olivier Windisch, Christophe Iselin, Daniel Benamran

**Affiliations:** Division of Urology, Geneva University Hospitals, 1211 Genève 14, Geneva, Switzerland

**Keywords:** Robotic surgery, Prostate cancer, Radical prostatectomy, Inguinal hernia, Prosthetic herniorrhaphy

## Abstract

**Purpose:**

Patients who undergo robot-assisted laparoscopic radical prostatectomy (RARP) may present concurrent or secondary inguinal hernia (IH). Surgical repair of IH simultaneously with RARP has been reported. We aimed to assess the long-term efficacy of concurrent prosthetic IH repair with RARP.

**Methods:**

Data for consecutive patients undergoing concurrent IH repair with RARP for localized prostate cancer at our institution between 2006 and 2017 were retrospectively analysed. Patients were matched based on age, BMI, and year of surgery, with patients undergoing RARP alone. IH repair was performed with a polyester mesh. Efficacy of IH repair was the primary outcome. Patient characteristics, perioperative data, recurrence and treatment were recorded.

**Results:**

A total of 136 men were included, 50% treated by RARP and concurrent IH, 50% by RARP alone. Mean age was 65 years (SD 6) and mean BMI 26.8 (SD 2.5). IH was diagnosed preoperatively in 42 patients (62%) or intraoperatively in 26 patients (38%). A total 18 patients (26%) had bilateral hernias and 50 patients had unilateral hernias (right 31%, left 43%). There was no significant difference between the two groups regarding perioperative data. The herniorrhaphy added 34 min to the operative time (p < 0.001). After a mean follow-up of 106 months [SD 38], 9 patients (13%) presented recurrence of IH, with a mean time to recurrence of 43 months [SD 35]. Age was significantly associated with IH recurrence (p = 0.0007).

**Conclusion:**

Concomitant IH repair and RARP appear to be a safe procedure with good long-term safety and efficacy, without significantly increasing morbidity.

## Introduction

Prostate cancer (PCa) is the second most commonly diagnosed cancer among men, with an estimated 1.4 million new cases in 2020 [1] with 70% of patients presenting with a localized disease [2]. RARP, first described by Binder in 2001 [3], is now part of the therapeutic armamentarium for treating localized PCa.

Approximately 15% of patients who undergo RARP develop a symptomatic IH within the first post-operative year [4]. Other series reported an almost fourfold increase in IH repair after radical prostatectomy (RP) compared to controls [5]. Regan et al. proposed that prolonged stretching of the rectus and transversalis fascia during retropubic exposure leads to weakening of the inguinal floor and ring [6]. According to Kyle et al., symptomatic IHs found post RP may represent subclinical IHs that manifest after surgery [7]. IH can be detected either at physical examination before RARP or incidentally during surgery. In the retrospective analysis by Fukuta et al. 20.4% of patients who underwent RP had evidence of hernias on preoperative computer tomography (CT) scans [8]. In the prospective study by Nielsen et al. up to 33% of patients had evidence of IH at the time of RP, 40% of which were bilateral [9].

The National Institute for Health and Care Excellence Guidelines (NICE) recommend open surgical repair for the treatment of primary unilateral IH [10]. However, in the last decades, the laparoscopic approach for IH repair has been shown to be safe and efficient and is now widely used in clinical practice [11]. Concomitant IH with prostatectomy was first performed by McDonald and Huggins in the late 1940s [12]. Nevertheless, routine repair of IH during RARP remains controversial, especially when a mesh is used, as there is a supposedly higher risk of mesh infection due to opening and drainage of the urinary tract.

The aim of the present study was to assess the long-term efficacy and safety of prosthetic inguinal herniorrhaphy concomitant with RARP.

## Material and methods

### Study population

A total of 136 patients, who underwent concurrent IH repair and RARP for localized PCa and at our institution between 2006 and 2017, were included in this monocentric observational study. A follow-up period of at least 5 years was ensured for all patients. Patients who underwent previous IH repair were excluded. For comparison, we identified a matched group based on age, body mass index (BMI) and year of surgery, who had undergone RARP alone. The study was approved by the local Ethics Committee.

### Data collection

Demographic and perioperative data were retrospectively gathered from patient files. Demographic data included: age, BMI, IH characteristics, preoperative PSA, clinical stage and histological grade of PCa. Perioperative data included: total operative time, hemoglobin drop, length of hospital stay (LOS) and 90-day postoperative complications reported according to the Clavien classification [13]. Oncological data comprised: pathological tumor stage (pTNM), histology, tumor grade, positive margins. Recurrence and treatment strategy were also recorded. Oncological follow-up of PCa was performed according to the European Association of Urology Guidelines, including a clinical and biological follow-up at regular intervals.

### Surgical procedure

All procedures were performed using the Da Vinci Robotic System Xi^®^ (Intuitive Surgical, Sunnyvale, CA, USA) by two surgeons with extensive previous experience of robotic surgery. Concurrent RARP and IH repair were performed by the same surgeon, using a transperitoneal approach. The peritoneum was divided from medial to lateral to the *vasa deferentia* on each side and the bladder was dropped down from the anterior abdominal wall along its avascular plane. This step was part of the usual RARP procedure and allowed direct visualisation of the hernia. The hernia sac was dissected free, as proximally as possible. IH repair using a polyester mesh (Parietex^®^, Medtronic or Proceed^®^, Ethicon) was performed as the last step of the surgery, after lymphadenectomy, prostatectomy and vesicourethral anastomosis. The mesh was cut into a rectangle shape with smooth-edged corners, of 12 × 8 cm for unilateral herniorrhaphy, and 20 × 8 cm for bilateral hernia repair. It was then anchored with absorbable staples (Absorbatack^®^, Covidien) to the abdominal wall and with non-absorbable sutures near the iliac vessels. Care was taken to avoid the sensory nerves that course laterally to the iliac vessels. The peritoneum was not closed after placing the mesh. A drain was inserted at the end of the procedure. Sulfamethoxazole-trimethoprime was given as a prophylactic antibiotic once a day until the urinary catheter was removed 10 days after surgery.

### Outcomes

The primary outcome measure was efficacy of IH repair. Patients benefited from an interview and a directed clinical examination to detect IH recurrence at regular intervals during PCa follow-up. Secondary outcomes included perioperative and oncological data.

### Statistical analysis

Statistical analyses were performed with R (v.4.2.2) and Stata15^®^ (College Station, TX, USA). Continuous variables are reported as mean and standard deviation [SD]. Categorical variables are described as frequency and percentage. Univariate analyses were performed using Wilcoxon signed-ranked tests for continuous variables, and McNemar-Bowker test for categorical variables. The IH recurrence-free survival was represented graphically by a Kaplan–Meier curve. All statistical tests were two-sided with p < 0.05 considered as statistically significant.

## Results

Patient and tumor characteristics are summarized in Table 1. A total of 136 patients were included, 68 (50%) of whom underwent RARP with concurrent IH repair and a matched group of 68 patients (50%) who underwent RARP alone. Mean age was 65 years [SD 6], mean BMI 26.8 kg/m^2^ [SD 2.5] and mean preoperative PSA 9.8 ng/l [SD 9.5]. There was no significant difference between the two groups regarding age, BMI (p = 0.086, 0.702, respectively). Preoperative PSA was significantly higher in the group with RARP and concurrent IH repair (p < 0.001), but ISUP grade (p = 0.210) and prostate volume (p = 0.779) were similar in both groups.

**Table 1:** Clinical and tumour characteristics of 138 patients undergoing RARP with or without concurrent IH repair

	Overall	RARP alone n = 68	RARP + IH repair n = 68	p value
Age (years)	65 (± 6)	64.7 (± 5.4)	65.2 (± 6.6)	0.086
BMI (Kg/m^2^)	26.8 (± 2.5)	26.8 (± 1.3)	26.8 (± 3.3)	0.702
PSA (µg/ml)	9.8 (± 9.5)	8.5 (± 10.3)	11.1 (± 8.6)	< 0.001
Prostate Vol (mm^3^)	50.5 (± 24.3)	50.6 (± 22)	50.3 (± 26.5)	0.779
ISUP grade, n (%)				0.210
1	52 (38.2)	25 (36.8)	27 (39.7)	
2	37 (27.2)	21 (30.9)	16 (23.5)	
3	20 (14.7)	10 (14.7)	10 (14.7)	
4	18 (13.2)	7 (10.3)	11 (16.2)	
5	9 (6.6)	5 (7.4)	4 (5.9)	
Hernia classification, n (%)				
Direct			35 (76.1)	
Indirect			11 (23.9)	
Missing data			22	

Unless specified otherwise, values are mean (SD)

*BMI* body mass index, *PSA* prostate specific antigen, *ISUP* international society of urological pathology

In the group of patients with RARP and concurrent IH repair, IH were identified on the left side in 29 cases (43%), on the right side in 21 cases (31%) and 18 patients presented with bilateral IH (26%). Regarding the IH classification, direct hernia was found in 35 (76%) patients and indirect hernia in 11 (24%) patients. IH were diagnosed preoperatively in 42 patients (62%) and intra-operatively in 26 patients (38%).

Mean operative time was significantly higher in the RARP and concurrent IH repair group (34 min longer) than in the RARP alone group (p < 0.001). Mean post-operative haemoglobin drop was similar in both groups. Median LOS was 4 days [SD 1] in both groups. Regarding early post-operative complications, 5 patients (7%) presented Clavien grade I and II events (paralytic ileus n = 1, minor anemia n = 4) in the group of patients with concurrent IH repair and RARP. In the RARP alone group, Clavien grade I and II complications were reported in 4 patients (6%) (pubic bone osteomyelitis n = 1, minor anemia n = 3). No Clavien grade III or superior event was reported in either group. Perioperative data are summarized in Table 2.

**Table 2:** Perioperative data of 138 patients undergoing RARP with or without concurrent IH repair

	Overall	RARP alone	RARP + IH repair	p value
Length of stay (days)	3.8 (± 1.4)	3.6 (± 1.2)	3.9 (± 1.5)	0.278
Post-operative day 1 haemoglobin decrease (g/dL)	26.2 (± 8.6)	25.2 (± 8.2)	27.2 (± 9)	0.208
Operative time (min)	276.2 (± 59.1)	259.3 (± 53.6)	293.4 (± 59.7)	< 0.001
Complications, n (%)				
Clavien-Dindo I	7	3	4	–
Clavien-Dindo II	2	1	1	
Clavien-Dindo ≥ III	0	0	0	

Unless specified otherwise, values are mean (SD)

**Fig. 1:**
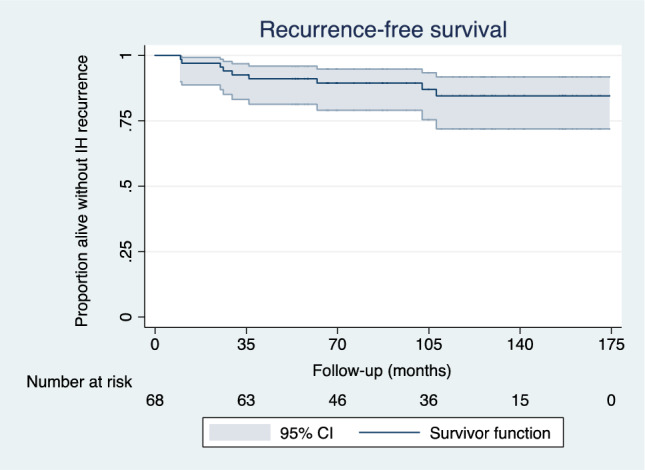
Inguinal hernia (IH) recurrence after RARP and concomitant IH repair

After a mean follow-up of 106 months [SD 38], 9 patients (13%) presented recurrence of IH, with a mean time to recurrence of 43 months [SD 35] (cf. Figure 1). Age was significantly associated with recurrence of IH (p = 0.0007). Secondary IH was treated by open Lichtenstein hernioplasty (88%) or by totally extraperitoneal repair (12%). No scrotal hematoma, seroma formation or mesh infection was reported during the follow-up period. In the combined procedure group, seven patients (11%) relapsed, six of them having new IH repair surgery. The last patient was asymptomatic, and no surgery indication was retained. Of note, 5 patients (7%) in the RARP alone group presented a clinically uncomplicated IH during the follow-up period (two on the left side, two on the right side, one bilateral). These patients were treated surgically using an anterior approach without any further complications. The mean time to diagnosis of recurrence was 75 months [SD 69].

Regarding oncological outcomes, 29% (40) of all patients presented a biochemical recurrence with a proven local recurrence during the follow up period. In the concurrent IH repair and RARP group, recurrence rate was 37% (25) with a mean time to relapse of 213 months, and in the RARP alone group, it was 22% (15) with a mean time to relapse of 189 months. All patients underwent systemic treatment, radiation therapy or a combination of both.

## Discussion

The current study assessed the long term functional and surgical outcomes of patients who underwent concurrent IH repair and RARP in a single academic centre. The results of our study underline that concurrent IH repair and RARP seem to be a safe procedure with good long-term efficacy. To date, this is the longest follow-up study regarding this combined procedure using a transperitoneal approach and a coated mesh. Over a mean follow up of over 8 years, treatment of an IH at the time of a RARP procedure shows very good overall efficacy, with no additional morbidity in patients treated surgically for localised prostate cancer.

Rates of IH recurrence after primary IH repair range from 0 to 25% for laparoscopic hernia repair and from 0 to 8% for the Lichtenstein technique on a 5 year follow-up [14]. In our study, recurrence rate of IH was 13%, similar to the recurrence rate of primary treated IH alone, confirming the efficacy of such a combined procedure. When considering studies that report concurrent IH repair and RARP, recurrence rates of IH were close to zero. In fact, Finley et al*.* reported an IH recurrence rate of 1.3% after 12 months of follow-up, while other studies found no recurrence at all [4, 9, 15]. Noteworthy, the median follow-up in these studies was considerably shorter than ours, which reached 106 months. We reported a mean time to recurrence of 43 months, which is a timeframe longer than most overall follow-up period published before in this setting. Therefore, it seems natural that our series of combined RARP procedure and IH repair would show increased IH recurrence.

Regarding complications, we report no mesh infection, clinically significant hematoma, seroma formation or bowel adhesion. Additionally, no Clavien complications grade 3 or higher were reported in both groups. In our centre, all bacteriuria, symptomatic or not, are preoperatively treated after antibiogram reception. Intraoperatively, vesicourethral anastomosis is tested (120 cc NaCl 0.9% intravesical instillation) looking for eventual leakage and prevent contact between urine and the mesh that will be used for IH repair. We used double-faced coated mesh in order to avoid small bowel adhesions and fixed it to the abdominal wall, which prevents not only migration but also the presence of dead spaces and potential seroma formation.

It is evident that adding IH repair during a RARP procedure will prolong total operative time. Previous studies report an additional 10–30 min to total operative time [16], which is in line with our results. The combined IH repair and RARP procedure is still considerably shorter than either surgeries performed independently [17]. Furthermore, the added operative time does not seem to increase blood loss, add any post-operative complications, or extend hospital length of stay, confirming the safety of the combined procedure.

Performing IH repair during the RARP procedure allows a privileged anatomical access to the inner inguinal ring, which favours a repair procedure using minimally invasive techniques. This is known to cause less pain and to reduce the length of stay [18]. Since there is an increased risk of symptomatic IH after RARP [19], the combined procedure can prevent infra-clinical IH from becoming symptomatic afterwards. Furthermore, inguinal repair in a previously operated site can be a more complex procedure [20]. Performing IH repair simultaneously with RARP can therefore avoid an ulterior complex laparoscopic IH repair or an open anterior repair, which avoids the risk of losing the benefits of minimally invasive surgery. Moreover, early elective treatment of IH has shown to be cost-effective, allows for faster recovery with less pain, decreases complications and recurrence rates, and shortens hospital LOS [21].

In the general population, cumulative lifetime IH incidence is up to 42% with male gender, age, low BMI, and history of prostatectomy being amongst the main risk factors [18]. In the pre-robotic era, Rabbani et al. described age, lower BMI, bladder neck contracture and previous IH repair as independent predictors of IH after RP [22]. More recently, a study including 693 patients undergoing RARP showed that lower urinary tract symptoms with a 15 or greater IPSS (International Prostate Symptom Score) was a risk factor for developing an IH after RARP [23]. Furthermore, factors such as age, lower BMI and lower subcutaneous fat mass have also been identified as risk factors for IH occurrence after RARP in another recent study [24]. In our cohort, age was the only predictor for IH recurrence.

The overall recurrence rate of PCa in our cohort is similar to previous published series with long term follow-up after treatment of localised PCa [25]. This underlines that the repair of IH during RARP should not affect oncological outcomes.

Our study has some limitations, including the small population size and its monocentric retrospective nature, even though data were recorded prospectively. In addition, the external validity of this study is limited since it only included men and therefore these data are not applicable to women undergoing concurrent IH repair and another minimally invasive pelvic procedure. Finally, since none of the included patients had previously undergone hernia repair, conclusions about recurrent IH cannot be made based on our findings.

Although concomitant IH repair and RARP still lack level 1 evidence to become standard practice, this combined procedure is becoming increasingly accepted due to available data and recent published reports [15, 26, 27], and should be considered rather safe and effective on the long term.

## Conclusion

Concurrent IH repair and RARP appears to be a safe procedure with good long-term safety and efficacy, without increased complications or morbidity. Particular attention should be given to the risk of IH recurrence on the long term and patients should be informed accordingly.

## Data Availability

The data that support the findings of this study are not openly available due to local ethical restrictions but can be requested from the corresponding author.
